# Home care experience and nursing needs of caregivers of children undergoing congenital heart disease operations: A qualitative descriptive study

**DOI:** 10.1371/journal.pone.0213154

**Published:** 2019-03-14

**Authors:** Zhi Hong Ni, Hai Tao Lv, Sheng Ding, Wen Ying Yao

**Affiliations:** Children’s Hospital of Soochow University, Suzhou, China; University of São Paulo, BRAZIL

## Abstract

**Aims and objectives:**

To explore the home care experiences of caregivers taking care of CHD children before and after cardiac surgery.

**Background:**

Despite the prevalence of congenital heart disease (CHD) in childhood, little is known about the experiences and impacts on the children and their caregivers after CHD diagnosis and surgery. Such knowledge is needed for meaningful support.

**Design:**

A qualitative descriptive study.

**Methods:**

Twenty-two caregivers of CHD children undergoing cardiac surgery participated in semi-structured interviews at a University Children’s Hospital in China. Data were collected by an experienced and trained interviewer. Qualitative content analysis was chosen to describe the experiences of the caregivers.

**Results:**

Caregivers of CHD children experienced significant demands. After the children underwent their CHD operations, the caregivers experienced complex psychological feelings and excessive stress impacting upon theirlives. In addition, caregivers constantly adapted their roles with self-fulfillment in caring activities.

**Conclusions:**

CHD surgery has a major impact on the emotions and daily lives of children and their caregivers. This study offers a framework for understanding the importance of actively listening to caregivers so coping strategies can be implemented.

**Relevance to clinical practice:**

Theexperiencesdescribed in this study contribute to a better understanding of the needs of caregivers whose children underwent CHD operations. They also provide valuable information to professional medical care staff that developfuture nursing assessments.

## Introduction

Congenital heart disease (CHD) is definedas having defect(s) (present since birth)in the structure of the heart.CHD includes several types of structural heart defects that develop prenatally, includingventricular septal defect (VSD), atrial septal defect (ASD), patent ductus arteriosus (PDA), and Tetralogy of Fallot (TOF).The worldwide prevalence of CHDis estimated at 1.35 million annually [1], and the incidence of children born with CHD is 1% [2], with over 100,000 new cases in China each year [3].With the development of medicine, when women are 18–20 weeks pregnant, fetuses can be screened for congenital heart disease under cardiac ultrasound. If a fetus has complex congenital heart disease, pregnant women can choose to terminate pregnancy at this stage.However, the detection rate of complex congenital heart disease by prenatal ultrasound screening is less than 50%.

CHD is the leading cause of newborn impairment andmortality in China [4]. Surgical intervention remains the mainstay treatment for CHD; however, corrective surgery is considered high risk and appropriate postoperative care is crucial for surgical wound healing, recovery, and survival.Ten percent of CHD children need corrective surgery, and the long-term survival rate after surgery is 90%. However, 50% of children with tetralogy of Fallot die within 3 years of age without surgery [4]. The patient is hospitalized in the cardiology department, andafter the operation, the child enters the intensive care unit without parental care. A few days later, the child enters the ward with parental care. Hospitals have specialized nurses to support these children and their families. After hospitalization, the cost of medical treatment is a large burden on families; 42% of parents have to bear the high cost of medical treatment in China due to differing availability and cost of medical insurance. Following surgery, CHD children are often discharged too early with incomplete recovery and continuing pain [5]. All of these circumstances cause significant distress and burdens for the parentsand other caregivers of the pediatric patients [6].

Spijkerboer [7]reported that parents of children who underwent corrective surgery for CHD often experienced negative emotional reactions and required ongoing home care support. Moreover, the psychological pressure experienced by the parents of CHD children was much higher compared to that caused by other chronic diseases [8]. Furthermore, the negative mental state of those parents not only affects their own health, but also impacts upon the rehabilitation and quality of nursing for their children. To better provide care for CHD children, parents need certain levels of postoperative knowledge, including how to understand children's medical information [9], reduce anxiety, help children, and get support and comfort [10]. In addition,health care professionals need to provide parents with disease management and treatment information, disease knowledge, regular ties with hospital, shared experiences and feelings, and home care visits after cardiac surgery [11].

More than 90% of caregivers believe that more support for heart patients is necessary, and more than 86% of caregivers believe that the intervention should be completed by cardiac healthcare professionals [12]. Our study aimed to explore the home care experiences of caregivers taking care of CHD children before and after cardiac surgery. Health care providersneed to completely understand the feelings and nursing needs of these caregivers and provide them with timely help and support to prevent negative emotions. In addition, medical professionals should improve the skills of caregivers and promote the rehabilitation of children.

## Methods

### Ethical considerations

This study was approved by the Ethics Committee of Children’s Hospital of Soochow University in Suzhou City, Jiangsu Province, China (Approval #2011002). The interviewees were informed of the purpose, methodology, content, and significance of this study. The caregivers had the right to participate in or leave the study even after signing the informed consent. Measures were established to provide confidentiality. We deleted any information that revealed the identity of the participants in the transcripts, and also assigned pseudonyms to all of the subjects who participated in this study.

### Design

This qualitative descriptive study was conducted between January 2016 and June 2016 via semi-structured, open-ended interviews to study the home care experiences and needs of caregivers of CHD children undergoing cardiac surgery. A total of 22 caregivers were enrolled in this study through purposive sampling. These caregivers, including 17 mothers and 5 fathers, were aged between 23 and 40 years. A convenience sample of children diagnosed with CHD and treated in the Department of Cardiac Surgery Department at Children’s Hospital of Soochow Universityduring the study period (January 2016–June 2016) was included. The Cardiac Surgery Department is a standalone cardiac center; 200 paediatric cardiac surgeries are performed a year by 7 cardiologists and 5 surgeons. The patients were supported by a multidisciplinary team of cardiologists, nurses, nurse specialists, psychologists, social workers, and dietitians.

The inclusion criteria for these CHD children and caregivers were: (1) children who accepted cardiac surgery for CHD treatment; (2)children older than 1 month but less than 60 months; (3)caregivers who were the children's parents; (4) caregivers who had normal cognition and expression ability; and (5) caregivers who voluntarily participated in this study and signed informed consent. The exclusion criteria for children and caregivers were: (1) caregivers who had a mental illness; and (2) children who had heart, lung, or brain functional failure or other serious complications.

Study data were continuously collected until no new events emerged. Thus, data saturation was achieved [13]. The demographic data of the children are summarized in Table 1. The demographic data of the caregivers are summarized in Table 2.

**Table 1:** Demographic data of the children with congenital heart disease.

Variables	n	F (%)
Gender		
Male	13	59.1
Female	9	40.9
Age (months)		
2–20	13	59.1
21–40	5	22.7
41–60	4	18.2
Residence		
City	8	36.4
County	14	63.6
Only child		
Yes	17	77.3
No	5	22.7
CHD type		
VSD	8	36.4
ASD	5	22.7
PDA	5	22.7
TOF	4	18.2

**Abbreviations:** congenital heart disease (CHD), ventricular septal defects (VSD), atrial septal defects (ASD), tetralogy of Fallot (TOF), patent ductus arteriosus (PDA).

**Table 2:** Demographic data of the caregivers.

Variables	n	F (%)
Education		
Middle School	7	31.8
Junior College	9	40.9
University	6	27.3
Income (yuan)		
<4000	5	22.7
4000–6000	11	50.0
>6000	6	27.3
Occupation		
Unemployed	3	13.6
Company worker	8	36.4
Agricultural worker (poor)	6	27.3
Office clerk	5	22.7
Caregiver		
Mother	17	77.3
Father	5	22.7

### Data collection

In this study, we developed a semi-structured interview after consulting six pediatric cardiac nurses and referring to systematic literature reviews [14–16].First, we conducted a preliminary interview of five caregivers. Then, we modified the interview outline on the basis of the outcome of these preliminary interviews; however, this pilot interview was not included in the data collection and analysis.

The final interview outline included the following information: (1) what the caregivers knew about the CHD operation procedures; (2) how they knew about the CHD operation procedures; (3) how they felt when they learned that their children had to undergo operations; (4) whether the nurses told them everything they needed to know and did they want to know anything else about the operation; (5) how they felt during the home care process; (6) what was the most difficult problem they encountered during the home care process; (7) how they solved those difficulties they encountered; (8) what was the most significant help they needed; (9) how they arranged their daily lives; and (10) whether their relatives supported them.

The caregivers’ responses were recorded throughout the interview sessions conducted 1 month after CHD children had undergone cardiac operations. Each interview session lasted 30–40 minutes.

### Data analysis

We performed qualitative content analysis of the data [17]. The interviews were transcribed verbatim, and the notes were then compiled. The second investigator read the transcripts twice to become familiar with the data prior to the development of words and phrases describing the home care experiences of caregivers of CHD children. Thereafter, the first and fourth investigator read transcripts of all interviews. The investigators extracted sentences containing information about home care experiences after the operation. The process continued with the completion of coding sheets, data grouping, category creation, and finally abstraction of categories. The various descriptions were placed under corresponding codes. The investigators worked together several times categorizing data. Thereafter, they segregated the data into five main categories. Finally, they went back to the caregivers of CHD children who participated in this study and validated their findings. All the interviewees agreed that the investigators had presented accurate results.

### Rigor

In this study, we took measures to ensure the credibility, transferability, consistency, and confirmability of results according to a previous study [18]. To increase credibility, we included all possible considerations and representations of study subjects who were chosen according to their age, sex, and level of education.

Before conducting this study, we developed a trustworthy and friendly relationship with all participants. To increase transferability, all caregivers were interviewed by the same interviewers who remained neutral and encouraged the interviewees to clearly express their feelings. Furthermore, the investigators objectively recorded the tone, intonation, and nonverbal expression of study subjects. Consistency was demonstrated by providing detailed descriptions of all phases of the analysis process. The interview duration and the number of interviews were extended after considering the actual situation. To increase the confirmability of data obtained from the actual content of interview, we included two investigators who had either very limited or no clinical experience with CHD children.

## Results

Analysis of the data identified five main themes, and each theme was supported by verbatim quotes from the study participants. The five themes are discussed below in the following order: (1) excessive mental burden, (2) under pressure and agony, (3) the impact on personal life, (4) adapting roles constantly, and (5) self-fulfillment in caring activities (Table 3).

**Table 3:** Description of the generic and main categories.

Generic categories	Main categories
Frightened and restless	Excessive mental burden
Remorse and guilt	
Fear of disclosure	
Physical strength overdraft	Under pressure and agony
Heavy economic burden	
Social impact	The impact of personal life
Break the good life	
Forget oneself	
Seek knowledge and help	Adapting roles constantly
Integrated care role	
Self-affirmation	Self-fulfillment in caring activities
Get satisfaction	

### Theme 1: Excessive mental burden

#### Frightened and restless

At first, the ability of the caregivers of CHD children to withstand sudden life changes was poor, which affected their mental health to a great extent. Because of China’s one-child policy, most CHD children were the only child in the family, and19 caregivers of these 22 believed that the child was significant to the family.

There is a CHD child in the family, just like a stone pressed in the heart, and it is difficult to relax. I've never had sound sleep since my baby underwent surgery. When I took a nap, I was often awakened by nightmares. (Caregiver #2)

Sometimes I went out and bought something, I always worried about the child at home. I am afraid he will have sudden difficulty breathing, and be rushed to hospital for treatment. (Caregiver #6)

#### Remorse and guilt

Somecaregiversappeared to suffer from feelings of remorse and uneasiness due to their child having CHD.Five caregivers blamed themselves for their children’s illnesses.

When I was pregnant at an early stage, I had very bad cold with a fever, and I took a lot of medications. My child obtained this disease because of that. The doctor said the some medications may cause congenital defects of embryos. (Caregiver#5)

When I was pregnant, my body was not good, therefore my child got the CHD. If I had known the child was ill, I shouldn't be pregnant. Now my little kid suffered so much. (Sigh) (Caregiver #11)

#### Fear of disclosure

Caregivers of CHD children were often reluctant to disclose their children’s condition to colleagues or friends for fear that their children and the entire family would receive discrimination thus affecting the children's future growth, both psychologically and physiologically. Seven caregivers believed that information about cardiac surgery should be kept confidential.

I take the child to the hospital, and I do not want to let others know. Sometimes when I met acquaintances in the hospital, I said that the child is only having a cold, just a minor illness. (Caregiver #7)

My child underwent heart surgery, but I didn't even tell my closest relatives and friends. If the information spreads out, other people will look at my children in a different way, which will affect his psychological health. CHD children who go to school and hunt for jobs in the future may be discriminated against. (Caregiver #4)

### Theme 2:Under pressure and agony

#### Physical strength overdraft

CHD children who underwent surgery sometimes had difficulty breathing and feeding, and cried uneasily. Caregivers had to perform a lot of manual labor, including feeding children, changing diapers, administering medicine, and bathing, and they often felt powerless. Seven caregivers felt physically and mentally exhausted and physically overdrawn.

I'm busy all day,no time to rest. Sometimes I only slept 2–3 hours each night, and I can't stand it anymore, but I cannot break down, because my child is so small.What can he do if I break down? (Caregiver #9)

I am just like a machine, taking care of my child from morning till night, including feeding milk and helping taking medicine. It is difficult for him to drink 30 ml of milk at one time; he needs to spit some out; bedding and clothing are often wet, so I must change his clothes, otherwise he will catch a cold again, which will exacerbate his illness. I think I'm going to break down. (Caregiver #1)

#### Heavy economic burden

For the family of a CHD child, the economic pressure is very great. Indeed, the cost of cardiac surgery is as high as 6000 yuan (US$10,000), and much money is spent on monthly visits to the hospital. In addition, dispensing and inspection expenses are also very large. Six caregivers believed that the children’s surgery had a strong impact on the family’s budget.

I have to take care of my child all the time, so I have no time to do housework; I have to hire an hourly employee who is paid 300 yuan US$50 per day. I have no work now, and our family life depends on my husband. His salary is only US$2000 per month, and I am almost unable to pay the medical fees. (Caregiver#19)

My family income is low, and we spent all our money on my son. I also feel embarrassed borrowing money from relatives and friends. Since the child's illness, the family's money has been used up. I hope our child could get better soon. (Caregiver #7)

In order to provide the child with appropriate medical treatment and ensure his medical fees are paid, I save every penny. Since he became ill, I have not bought a new dress for myself, and never bought anything expensive. (Caregiver #8)

#### Disharmony in family relations

Caregivers of CHD children often have friction with other family members because of the family chores and the heavy burden of home care. Moreover, the child is young, cannot communicate with parents, and cannot offer comfort. Caregivers lack emotional support and suffer great pain themselves. Five caregivers could not cope with family conflicts appropriately.

I take care of the child every day, and have no-one to discuss anything with. The child cannot talk and just cries. My husband works late every day, so he has no time to take care of our baby. I am so depressed! (Caregiver #3)

It is so hard for me to take care of the baby every day. I don't know if he will ever fully recover. My mother-in-law often quarrels with me about the child's things, and my husband always blames me for not communicating well with my mother-in-law. It's really annoying! Sometimes I feel so lonely, so desperate. (Caregiver #16)

### Theme 3:The impact on personal life

#### Social impact

Caregivers of CHD children usually had no space and time for themselves, anddid not participate in social activities. Five caregivers gave up their original responsibility and role in the work.

Ever since I heard that children had the disease, I have no mood to do my own thing. Originally,I planned to pursue postgraduate studies in 2 years and look for new career development opportunities, but the child is not well and I don't have time and energy to learn. (Caregiver #7)

Some caregivers had almost lost touch with the outside world.

In order to take care of the sick child, I never participate in my company's dinners and activities, let alone travel and vacations. My child's body is weak and he cannot stay alone without me. (Caregiver #2)

Caregivers live in the world of their children every day and do not have their own world.

I had a very good plan for my career and my future; now everything has been destroyed. I can't find my own value. I just want my children to get better soon. That's my only wish now. (Caregiver #12)

#### Breaking the good life

Many young parents had an optimistic vision and plan for family and life, but when the child was diagnosed with CHD, all hope vanished like soap bubbles.

I had planned to save much money so that I may travel abroad with my family for a holiday. Now that my child underwent cardiac surgery, the plan was ruined. (Caregiver #4)

I planned to buy a new house in 2 years, so that my retired parents may come and live in it. Now all the money has been spent on the child’s operation; I can't afford to buy the house anymore. (Caregiver #8)

#### Forgetting self

Caregivers of CHD children usually devoted all their energy and time to care for child and often neglected their own health needs.

I moved around him every day. Sometimes I think I live for him. I'm not feeling well and I often ignore it. When my child feels uncomfortable, I don't delay a minute; I take him to the hospital at once, and visit the doctor on time every week. (Caregiver #10)

Caregivers sometimes achieved a state of selflessness.

My friend said, “you take care of the child every day except yourself. You didn't do that before!” Sometimes I feel I'm really forgetting myself. (Caregiver #15)

### Theme 4: Adapting roles constantly

#### Seeking knowledge and help

When caregivers learned that their children had CHD, it came as a ‘bolt from the blue’ for many of them. However, over time, they gradually accepted the reality.

I couldn’t accept the reality at first; we don't have this hereditary disease in our family. What's the matter? However, after many doctors diagnosed my child, I admitted this fact. Now I have searched a lot of related medical knowledge online, and learned a lot of postoperative rehabilitation knowledge, which is very helpful to me for taking care of my baby. (Caregiver #13)

Seven caregivers perceived their lack of knowledge and ability and expressed their desire to consult and study with professionals.

When I was in the hospital, I consulted the doctors and nurses about some of the knowledge of cardiac surgery, and the nurses were very patient with me. (Caregiver #3)

I hope professionals will continue to help and care for us. I will do my best to take care of my child so that he can recover soon. (Caregiver #9)

#### Integrated care role

While taking care of CHD children, caregivers gradually acquiesced to their roles.

When I was young, my parents doted on me and I never did any housework. My parents are old now, their health is not good, and they can't help me anymore. Now I am the pillar of the family; I must be strong; I'm going to prop up my family. (Caregiver #21)

I have to take good care of my child. He's so weak. I want to protect him. I know I am the backbone and the hope of this family, and I can't count on anyone else. It's my duty. (Caregiver #13)

### Theme 5: Self-fulfillment in caring activities

#### Self-affirmation

Caregivers of CHD children often bore a heavy burden on their body and mind, but at the same time they gained similar happiness to other people.

I feel as a mother especially great; whether or not society needs me, whether or not my company needs me, my child definitely needs me. (Caregiver #20)

Taking care of children made the caregivers give up a lot, but also made them become more mature. Six caregivers affirmed the value of their existence.

I never felt the responsibility of being a mother like I do now. Now I think the most successful thing is that the baby smiles at me. I think it feels precious. (Caregiver #17)

#### Get satisfaction

A very important reason that caregivers of CHD children try their best to take care of their children is to fulfill their responsibilities. Moreover, they achieved a sense of satisfaction when the children gradually recovered.

I am the father of my child, and my child is the continuation of my life. Thus, it is my duty to take care of my child. I see the future in him. (Caregiver #14)

When the baby calls me ‘mum’, I feel so happy. When I saw him recovering gradually, I felt it was worthwhile to pay a lot more [money]. (Caregiver #22)

## Discussion

We conducted a qualitative descriptive study via interviews on the home care experiences of 22 caregivers of CHD children who had undergone cardiac surgery, and found that the postoperative care and care pathways of children with different types of CHD were similar. The diagnosis and surgical treatment of CHD children was a difficult process for the caregivers, and they were constantly under great pressure. Therefore, their needs and feelings should become the focus of the health care professionals’ attention.

### Diagnosis period (preoperativeperiod)–shock and denial

CHD is a severe illness, and can lead to heart failure and even death if no timely surgical treatment is given. Therefore, if children suffer from CHD, it is a huge blow to a family, and the impacts on both the child and the family are profound. In the present study, we found that caregivers were most strongly aware of their emotional reactions and psychological distress (including shock, denial, and hopelessness) when they first learned that their children had CHD, which was consistent with other reports [6]. At an early stage, parents were not able to accept any explanation, and did not think they were true. Additionally, at this time, they usually took the child to heart specialists looking for an alternative diagnosis. The intensity and timing of each caregiver’s experience were different. Parents usually experienced this great pressure for approximately 2–3 months. Then, most parents calmly accept the fact that their children had CHD.

### Operational period–inner fear and suffering

It is expected that CHD children need to wait for the appropriate time for surgery because they need to have good physical condition and nutritional status in order to tolerate the surgical trauma. During thoracic surgery, CHD children undergo general anesthesia, tracheal intubation, mechanical ventilation, and chest closed drainage, and these procedures are a grave test for each of those CHD children. Moreover, their caregivers were filled with inner fear and suffering; they felt powerless. Lawoko et al [19] investigated the anxiety level of parents of CHD children who underwent open chest surgery, and found that these parents were generally anxious. Cardiac surgery was associated with high risk and great trauma, and the efficiency of the cardiac operation as part of the CHD treatment as well as the risk of complications were the caregivers’ biggest concerns [20].

The degree of postoperative rehabilitation of the children had a direct impact on the emotional changes of the caregivers. If a child recovered well after the operation, the caregiver would usually experience a stable mood and inner joy. In contrast, once the child had severe complications after surgery, caregivers showed a high level of anxiety [21]. The most significant support caregivers needed was medical care; they hoped that the health care professionals would provide the best service and care for their children. Hence, it is important for health care professionalsto give parents all necessary information about their children as timely and accurately as possible.

### Convalescence period (postoperativeperiod)–worries about the future

After entering arelatively stable period, the caregivers began to worry about their children’s education, employment, and marriage in the future. After surgery, CHD children also faced long-term cardiac function recovery, scar healing, and other issues, and they were taken to the hospital for regular follow-up visits and monitoring of cardiac function [14]. At this stage, thehealth care professionalsshould pay attention to the general health status of CHD children and caregivers and compile the corresponding health education plan and nursing implementation guide.

When parents learned that their children suffered from CHD, their previous expectation of having a healthy baby was destroyed, and they were plunged into despair.Pye et al [22]showed that when parents learned their children suffered from CHD, regardless of whether CHD was serious and whether the child was suffering from other diseases, parents experienced anxiety and sadness, and were unwilling to accept the reality. Indeed, they usually showed emotional distress including depression, despair, and anger. In line with the above findings, in the present study, we found that caregivers felt regretful and inferior for failing to have a healthy child, and almost all caregivers blamed themselves for their children’s suffering from CHD, and were extremely self-critical. These parents also became very confused because they worried about possible failure in taking good care of their children, which was consistent with another report [23]. Thus, caregivers suffered from a great deal of psychological pressure, and they adopted different coping styles. Since caregivers had different understanding of difficulties and setbacks, their coping abilities and styles were different as well.Mahle et al [24]believed that parents' positive coping techniques can provide children with very good psychological support and promote their rehabilitation. Therefore, health care professionals should pay close attention to the mental health of caregivers and give them extra emotional support. In the early stages of admission, health care professionals should establish friendly relations with caregivers, communicate openly, and encourage them to express their fears, and teach them how to adjust their thinking. Moreover, during the communication process, health care professionalsshould learn to understand parents’ coping styles and encourage them to adopt positive attitudes and methods to cope with stressful life events. The constant burden of home care puts caregivers in a state of work overload. In this study, since all the children were infants, caregivers had to live with a heavy burden of care, including night time feeding, and pacifying the crying children to enable them to sleep. As a result, caregivers did not have a sufficient amount of time to take adequate rest. Consequently, these excessive physical demands negatively affected their health [25]. In addition, caregivers spent nearly all of their time taking care of children, resulting in termination of work, lack of social roles, and fewer opportunities to participate in recreational activities. In this chronic state of stress, good health was difficult to achieve for caregivers.

Hence, health care professionalsshouldinvestinefforts to reduce caregivers’ stress, encourage other family members to share burdens, and enhance the caregivers’ quality of life. Nurses, as social support personnel for sick children and their caregivers should provide empowerment-based healthcare education to parents of CHD children, improve parents’ caring knowledge and skills, which will eventually result in better recovery outcomes for the patients [26]. In the present study, we found that caregivers were more concerned with the surgical arrangements before the operation, and how to take good care for children after the operation. Nurses should inform caregivers about the medical treatment, surgery, and nursing information, and promptly appease any anxiety and confusion. In addition, nurses should offer lectures on nursing knowledge in the ward, establish a follow-up system, and select appropriate ways to answer questions from caregivers of different educational backgrounds. Caregivers not only focused on the children’s operations, but also worried about their children’s futures. Moreover, most caregivers were concerned about their children’s weaknesses as well as any social discrimination they would probably face in the future so they often concealed their children’s condition to others. In this regard, we hope that the general public will become more knowledgeable and empathic about CHD through publicity campaigns, thus reducing potential prejudices, and enabling a healthy and happy environment for CHD children.

Families of children with CHD cope differently depending on individual and familial factors beyond the severity of the child's condition. Recent research has shifted from an emphasis on the psychopathology of family to a focus on the resilience of families in coping with the challenges presented by a young child's condition [27]. The increasing number of studies on the relationship between psychological adaptation, parental coping and parenting practices and quality of life in families of children with CHD necessitates an in-depth re-exploration [28].Bettertargetedparent and family interventions are designed to enhance family coping. Families which have fewer psychosocial resources and lower levels of support may be at risk of higher psychological distress and lower well-being over time, for both parent and the child. Moreover, familial factors such as cohesiveness and adaptiveparentalcoping strategies are necessary for successful parental adaptation to their CHD child. The experiences, needs and ways of coping in families of CHD children are diverse and multi-faceted. A holistic approach to early psychosocial intervention should target improved adaptive coping and enhanced productive parenting practices in this population, which should lay a strong foundation for these families to successfully cope with future uncertainties and challenges at various phases in the trajectory of the child's condition.

## Study limitations

This study had some limitations. For example, we only recruited caregivers whose CHD children were aged between 1 month and 60 months. In a future study, we intend to expand the scope of study subjects and longitudinally interview the recruited caregivers. We also noted that five themes had some overlapping, which appeared to be inevitable in this study.

## Relevance to clinical practice

This study describes the home care experiences and needs of caregivers whose children underwent CHD operations. The experiences of caregivers providing care to CHD children are complex, Hence, we need to sufficientlytrain healthcare staff so that they identify the needsofcaregivers and provide them with targeted interventionthat meets their demands. The findings from this study highlight the need for more effective and individualized nursing intervention for CHD caregivers.

## Conclusions

In this study, we demonstrated that caregivers of CHD children who underwent cardiac surgery are under great psychological pressure during the home care rehabilitation period post operation. Health care professionals should try to fully understand the feelings of these caregivers, and provide them with planned and relevant medical guidance as well as related information on the surgery to enhance their confidence. To further strengthen the care and emotional support,health care professionals should develop care plans for both CHD children and their caregivers, and reduce the intensity of stress effectively so that caregivers can maintain their physical and mental health.

## Supporting information

S1 File CHD children’sevaluation questionnaire data. This file contains the data from the evaluation questionnaire.(SAV).
(SAV)
